# Rosuvastatin Upregulates RCOR1 to Repress C10ORF10 Transcription and Alleviate Oxidative Stress and Plaque Formation in Atherosclerosis

**DOI:** 10.1002/kjm2.70106

**Published:** 2025-09-09

**Authors:** Xin Fu, Chuang Liu, Ya‐Li Chen, Jie‐Lin Qin, Ting‐Ting Wang, Shan‐Shan Fu

**Affiliations:** ^1^ Department of Cardiology The First Affiliated Hospital of Zhengzhou University Zhengzhou People's Republic of China; ^2^ Department of Comprehensiveness The First Affiliated Hospital of Zhengzhou University Zhengzhou People's Republic of China

**Keywords:** atherosclerosis, C10ORF10, HVSMCs, RCOR1, rosuvastatin

## Abstract

Rosuvastatin (RVS) is an HMG‐CoA reductase inhibitor with lipid‐lowering properties. This study aims to investigate the role of RVS in plaque formation in atherosclerosis (AS) and its functional mechanism. ApoE^−/−^ mice were fed a high‐fat diet to generate a mouse model of AS. RVS treatment reduced serum levels of total cholesterol, triglycerides, and low‐density lipoprotein cholesterol in atherosclerotic mice, alleviated oxidative stress, and ameliorated lipid deposition, plaque formation, and fibrosis in the mouse aortic tissues. In vitro, it reduced reactive oxygen species and suppressed the proliferation and migration of oxidized low‐density lipoprotein‐challenged human vascular smooth muscle cells (HVSMCs). REST corepressor 1 (RCOR1) was identified as a target protein upregulated by RVS. It was found to repress transcription of decidual protein induced by progesterone 1 (DEPP1/C10ORF10) by binding to its promoter. Silencing of RCOR1 negated the AS‐ameliorating effects of RVS in mice and HVSMCs. However, the AS‐like symptoms in mice and HVSMC activity were suppressed by the additional C10ORF10 silencing. In conclusion, this study demonstrates that RVS alleviates oxidative stress and reduces atherosclerotic plaque formation by increasing RCOR1‐mediated transcriptional repression of C10ORF10.

## 1 Introduction

Atherosclerosis (AS) is a chronic, inflammatory condition affecting medium and large arteries, driven by lipid accumulation, and high levels of low‐density lipoprotein (LDL) contribute to vascular complications [1]. This retention promotes the uptake by scavenger receptors, triggering persistent infiltration of immune cells into the atherosclerotic plaque [2]. In advanced stages, plaque rupture can occur, leading to blood clot formation and acute cardiovascular incidents [3], significantly contributing to high rates of morbidity and mortality in both developing and developed nations [4].

The development of an intimal plaque involves a complex interplay of factors, including inflammation, lipid accumulation, vascular smooth muscle cell (VSMC) proliferation, endothelial dysfunction, and remodeling of the extracellular matrix [5]. VSMCs, along with endothelial cells and leukocytes, are key contributors to the progression of AS [6]. Under the influence of inflammatory conditions, oxidative stress, or other environmental triggers, VSMCs undergo a phenotypic shift from a quiescent, contractile state to an active, synthetic state [7]. VSMCs proliferate and migrate to the vascular intima, and these cells account for 30% to 70% of the total foam cells within the plaque, a finding that applies to both mice and humans [8].

Rosuvastatin (RVS) is a highly potent inhibitor of 3‐hydroxy‐3‐methylglutaryl coenzyme A (HMG‐CoA) reductase, known for its lipid‐lowering effects [9]. It has been proposed as a therapeutic strategy to decelerate AS progression by reducing levels of LDL‐cholesterol (LDL‐C) and total cholesterol (TC) to target thresholds [10]. Moreover, RVS has demonstrated the ability to suppress oxidative stress and inflammation in AS [11]. Oxidative stress is a critical driver in AS progression, elevating tissue levels of pro‐inflammatory cytokines and inducing irreversible pro‐atherogenic changes in the vessel wall, which increase arterial stiffness and reduce elasticity [12].

In this study, we identified REST corepressor 1 (RCOR1) as a potential target protein of RVS, and decidual protein induced by progesterone 1 (DEPP1/C10ORF10) as a likely target transcript of RCOR1 through integrated bioinformatics analysis. RCOR1 is known as a transcriptional repressor that recruits lysine‐specific demethylase 1A and histone deacetylases to chromatin, facilitating the removal of histone methylation and acetylation marks [13]. Furthermore, C10ORF10 has been shown to play a role in regulating the detoxification of reactive oxygen species (ROS), which contributes to oxidative stress‐induced damage [14]. However, the roles of RCOR1 and C10ORF10 in VSMC activity and AS progression remain unexplored. This study aims to determine whether RVS affects VSMC activity and AS progression through modulation of the RCOR1/C10ORF10 axis.

## 2 Materials and Methods

### 2.1 Animal Treatment and Modeling

The use of animals was approved by the Animal Ethics Committee of the First Affiliated Hospital of Zhengzhou University. All animal experiment procedures were conducted following the Guide for the Care and Use of Laboratory Animals (NIH, Bethesda, MD, USA). Eight‐week‐old ApoE^−/−^ mice (Strain code: 221; 20–25 g) and age‐matched C57BL/6J mice (Strain code: 219; 19–22 g) were purchased from Vital River Laboratory Animal Technology Co. Ltd. (Beijing, China). The mice were housed in specific pathogen‐free grade conditions in an environment at 23°C, 60% humidity, and a 12 h/12 h dark/light cycle and provided with free access to food and water.

The pAV[shRNA]‐EGFP‐U6‐based adenovirus for knockdown was purchased from VectorBuilder (Guangzhou, Guangdong, China), with a viral titer of 10^12^ VP/mL. After acclimatization for 1 week, the ApoE^−/−^ mice were injected with adenoviral vectors containing short hairpin RNA (shRNA) of RCOR1 and C10ORF10 (sh‐RCOR1 and sh‐C10ORF10, The shRNA sequences contained in the adenovirus are shown in Table 1), or the negative control (sh‐NC). Each mouse was injected with 100 μL of each adenovirus every 2 weeks. At the end of the experiment (12 weeks later), mice were euthanized by intraperitoneal injection of 150 mg/kg sodium pentobarbital, and aortic tissue was collected for gene expression analysis to confirm the efficiency of adenovirus‐mediated gene knockdown.

**TABLE 1:** shRNA sequence.

For mouse	
sh‐RCOR1	GAAGAAACAAACGGCAGTAATCTCGAGATTACTGCCGTTTGTTTCTTC
Sh‐C10ORF10	GTATCCTAGGTACTCTCTATTCTCGAGAATAGAGAGTACCTAGGATAC
For cells (human)	
sh‐RCOR1	CCCAATAATGGCCAGAATAAACTCGAGTTTATTCTGGCCATTATTGGG
For mouse and cells	
sh‐NC	CCTAAGGTTAAGTCGCCCTCGCTCGAGCGAGGGCGACTTAACCTTAGG

Abbreviations: C10ORF10, decidual protein induced by progesterone 1; NC, negative control; RCOR1, REST corepressor 1; shRNA, short hairpin RNA.

After the first adenovirus injection, the ApoE^−/−^ mice were fed a high‐fat diet (HFD) containing 0.15% cholesterol, 21% fat, 15.5% protein, and 62% normal diet for 12 weeks to establish the AS model. C57BL/6J mice in the normal group were fed a normal diet. For mice requiring RVS treatment, they were administered RVS (T1676, TargetMol Chemicals Inc., Shanghai, China) dissolved in saline solution intragastrically at a dose of 10 mg/kg/day, with an equivalent volume of normal saline (NS) used as a control regimen [15]. After 12 weeks, the mice were euthanized.

### 2.2 Database Search

Transcriptional differences between ox‐LDL‐treated (AS) and untreated (normal) human aortic endothelial cells were analyzed using the dataset in the GEO database (https://www.ncbi.nlm.nih.gov/geo/query/acc.cgi?acc=GSE137578). Using the online tool GEO2R (https://www.ncbi.nlm.nih.gov/geo/geo2r/), we analyzed the differential gene expression between the AS group (GSM4081920–GSM4081922) and the normal group (GSM4081917–GSM4081919). Benjamini & Hochberg was used to correct the *p*‐value, and differentially expressed genes were screened with an adj. *p*‐value < 0.01 as the threshold. A volcano plot of the differential expression analysis results was generated using Hiplot Pro (https://hiplot.com.cn/).

The online web search tool HumanTFDB (http://bioinfo.life.hust.edu.cn/HumanTFDB#!/) was used to download a list of human transcription factors, and the ChEMBL database (https://www.ebi.ac.uk/chembl/) was used to download potential targets for RVS. Jvenn (https://jvenn.toulouse.inrae.fr/app/example.html) was used for the intersection.

### 2.3 Sample Collection

After euthanizing the mice, fresh blood samples were drawn from the thoracic aorta and centrifuged at 1300 *g* for 10 min at 4°C to obtain serum. After blood collection, the thoracic and abdominal cavities of the mice were quickly opened to expose the heart. The cardiovascular system was perfused, and the aortic root and myocardial tissues were collected and either fixed in 4% paraformaldehyde or preserved at −80°C.

### 2.4 Cells and Treatment

Human VSMCs (HVSMCs, CD5004, Idraft BioTech., Shanghai, China) isolated from umbilical cord vessels at passage three were cultured in Dulbecco's modified Eagle's medium (11965118, Thermo Fisher Scientific, Rockford, IL, USA) supplemented with 10% fetal bovine serum and 1% penicillin/streptomycin in a humidified environment at 37°C with 5% CO_2_.

The shRNA‐EGFP/Puro‐U6 > vector‐based knockdown lentivirus (sh‐RCOR1/sh‐NC) and the pLV[Exp]‐EGFP/Puro‐EF1A > vector‐based overexpression lentivirus (OE‐C10ORF10/OE‐NC) were purchased from VectorBuilder, with a lentivirus titer > 10^8^ TU/mL. OE‐C10ORF10 contains the CDS sequence of C10ORF10 (NM_007021.4), and OE‐NC is an overexpression lentiviral vector without inserted sequences. The shRNA sequences contained in the lentivirus are shown in Table 1. HVSMCs were infected with lentiviral vectors for 48 h at an MOI of 10. Stable cells were selected using 3 μg/mL puromycin. After 72 h of puromycin screening, the cells were collected to confirm gene interference efficiency. Following this, the HVSMCs were pre‐incubated with RVS or Atorvastatin (AVS, T20765, TargetMol) at a concentration of 10 μM for 2 h and further incubated with 100 μg/mL ox‐LDL (S24879, Yuanye Bio‐Technology Co. Ltd., Shanghai, China) for 24 h [16].

### 2.5 Biochemical Tests

Mouse aortic tissues were weighed, clipped, and placed in centrifugation at 600 g for 10 min in saline. The supernatant was taken. The levels of TC (A111‐1‐1), triglycerides (TG, A110‐1‐1), and LDL‐C (A113‐1) in the collected serum samples were determined using the respective ELISA kits (all provided by Nanjing JianCheng Bioengineering Institute, Nanjing, Jiangsu, China).

### 2.6 Evaluation of Oxidative Stress Markers

According to the kit instructions, the levels of malondialdehyde (MDA, A003‐1‐2, Nanjing Jiancheng) and superoxide dismutase (SOD, A001‐3‐2, Nanjing Jiancheng) in the tissue homogenate of the mouse aorta were determined following the manufacturer's instruction manual.

The fresh proximal aorta was embedded in Tissue‐Tek OCT compound (4583, Sakura Finetek, Torrance, CA, USA) and frozen rapidly. The fresh tissue sections and the HVSMC slides were incubated with 10 μM dihydroethidium (DHE) (S0063, Shanghai Beyotime Biotechnology Co. Ltd., Shanghai, China) at 37°C in the dark for 30 min to analyze the levels of ROS. The images were observed under a fluorescent microscope.

### 2.7 Histological Assessment

Atherosclerotic plaque in the tissue sections was determined using hematoxylin and eosin (HE) staining, following the instructions of the HE staining kit (C0105S, Beyotime).

### 2.8 Reverse Transcription‐Quantitative Polymerase Chain Reaction (RT‐qPCR)

Total RNA was extracted from the mouse aortic tissue or HVSMCs using TRIzol (A2010A0402, BioTNT Biotechnologies, Shanghai, China), which was reverse‐transcribed into cDNA using the PrimeScript RT reagent Kit (RR037Q, Takara Holdings Inc., Kyoto, Japan) according to the manufacturer's instructions. Following this process, real‐time qPCR analysis was performed using ChamQ SYBR qPCR Master Mix (Q311‐02, Vazyme Biotech Co. Ltd., Nanjing, Jiangsu, China) in a StepOnePlus Real‐Time PCR System (4376600, Thermo Fisher Scientific). Relative gene expression was determined using the 2^−∆∆Ct^ method with glyceraldehyde‐3‐phosphate dehydrogenase (GAPDH) serving as the internal reference. The primers used are as follows: Human RCOR1 (F) 5′‐CCAGTAACCAGAAGCCTGTGAAG‐3′, (R) 5′‐AAGCCACCAGTTTCTCAGGAGG‐3′; Human DEPP1 (C10ORF10) (F) 5′‐GTGAGGTCTATATCTCGACTGGC‐3′, (R) 5′‐CCACTGAAACGTGCGGTGATGT‐3′; Mouse DEPP1 (C10ORF10) (F) 5′‐TCAGTGCTGGACAAGGTCACAG‐3′, (R) 5′‐CTGACGCAAAGAGAGCTGTCTC‐3′; Human GAPDH (F) 5′‐GTCTCCTCTGACTTCAACAGCG‐3′, (R) 5′ ‐ACCACCCTGTTGCTGTAGCCAA‐3′; and Mouse GAPDH (F) 5′‐CATCACTGCCACCCAGAAGACTG‐3′, (R) 5′‐ATGCCAGTGAGCTTCCCGTTCAG‐3′.

### 2.9 Western Blot (WB) Analysis

Total protein was extracted from aortic tissues and HVSMCs using radio‐immunoprecipitation assay lysis buffer (P0013C, Beyotime), and the protein concentration was measured using the bicinchoninic acid kit (P0012, Beyotime). Following this, the protein sample was separated using sodium dodecyl sulfate‐polyacrylamide gel electrophoresis and transferred onto polyvinylidene fluoride membranes. The membranes were blocked with 5% non‐fat milk at room temperature for 1 h and then incubated overnight at 4°C with primary antibodies against α‐SMA (1:1000, A7248, ABclonal Technology Co. Ltd., Wuhan, Hubei, China), RCOR1 (1:1000, A3568, ABclonal), C10ORF10 (1:1000, H00011067‐B01P, Thermo Fisher Scientific), and GAPDH (1:10,000, ab181602, Abcam), followed by incubation with HRP‐conjugated goat anti‐rabbit IgG (1:2000, ab6721, Abcam) or goat anti‐mouse IgG (1:5000, 31430, Thermo Fisher Scientific) at 37°C for 1 h. The protein bands were developed using enhanced chemiluminescence. The band signals were determined using ImageJ software.

### 2.10 Cell Counting Kit‐8 (CCK‐8) Method

According to the manufacturer's instructions (40203ES60, YEASEN Biotechnology Co. Ltd., Shanghai, China), HVSMC suspensions were seeded into 96‐well plates (10,000 cells per well) and incubated overnight. At 0, 24, 48, and 72 h, 10 μL of CCK‐8 reagent was added to each well and incubated at 37°C for 3 h. The OD at 450 nm was determined using a microplate reader.

### 2.11 Transwell Assays

A suspension of 5 × 10^4^ HVSMCs (300 μL) was added to the upper chamber of the 24‐well Transwell insert (CLS3378, Corning Glass Works, Corning, NY, USA), with 700 μL of medium containing 15% fetal bovine serum added to the lower chamber. After incubating under normal conditions for 24 h, the migrated cells were fixed with 4% paraformaldehyde and stained with 0.1% crystal violet (C0121, Beyotime) for 10 min. The number of migrating cells was calculated under the microscope.

### 2.12 Dual‐Luciferase Reporter Gene Assay

The promoter segment of C10ORF10 was inserted into pGL4.10 reporter vectors (E6651, Promega Corporation, Madison, WI, USA) and transfected with the control Renilla luciferase control reporter vector pRL‐TK (E2241, Promega) into HVSMCs stably transfected with sh‐RCOR1. After 48 h, the luciferase activity in cells was determined using the dual‐luciferase reporter gene assay kit (RG027, Beyotime).

### 2.13 Chromatin Immunoprecipitation (ChIP‐qPCR)

ChIP assays were performed according to the SimpleChIP Enzymatic ChIP Kit (9003, Cell Signaling Technologies, Beverly, MA, USA). HVSMCs were cross‐linked with formaldehyde at 37°C for 10 min, then quenched with glycine and quickly frozen. Chromatin was sonicated into 200–1000 bp fragments. DNA fragments were immunoprecipitated overnight at 4°C with the RCOR1 antibody (1:1000, A12845, ABclonal) or rabbit IgG (1:50, 2729, Cell Signaling Technologies). The immunoprecipitated complexes were collected. The DNA sample was eluted and purified; the enrichment of C10ORF10 promoter fragments was determined using qPCR analysis.

### 2.14 Statistical Analysis

Prism software (version 10.4.2, GraphPad, San Diego, CA, USA) was applied for data processing and bar graph generation. Measurement data, collected from no less than three independent experiments, are presented as the mean ± standard error of the mean. Comparisons were performed using *t*‐tests or using analysis of variance (ANOVA) followed by Tukey's post hoc tests, as appropriate. A *p*‐value of < 0.05 was considered statistically significant.

## 3 Results

### 3.1 RVS Treatment Ameliorates AS‐Associated Symptoms in Mice

**FIGURE 1:**
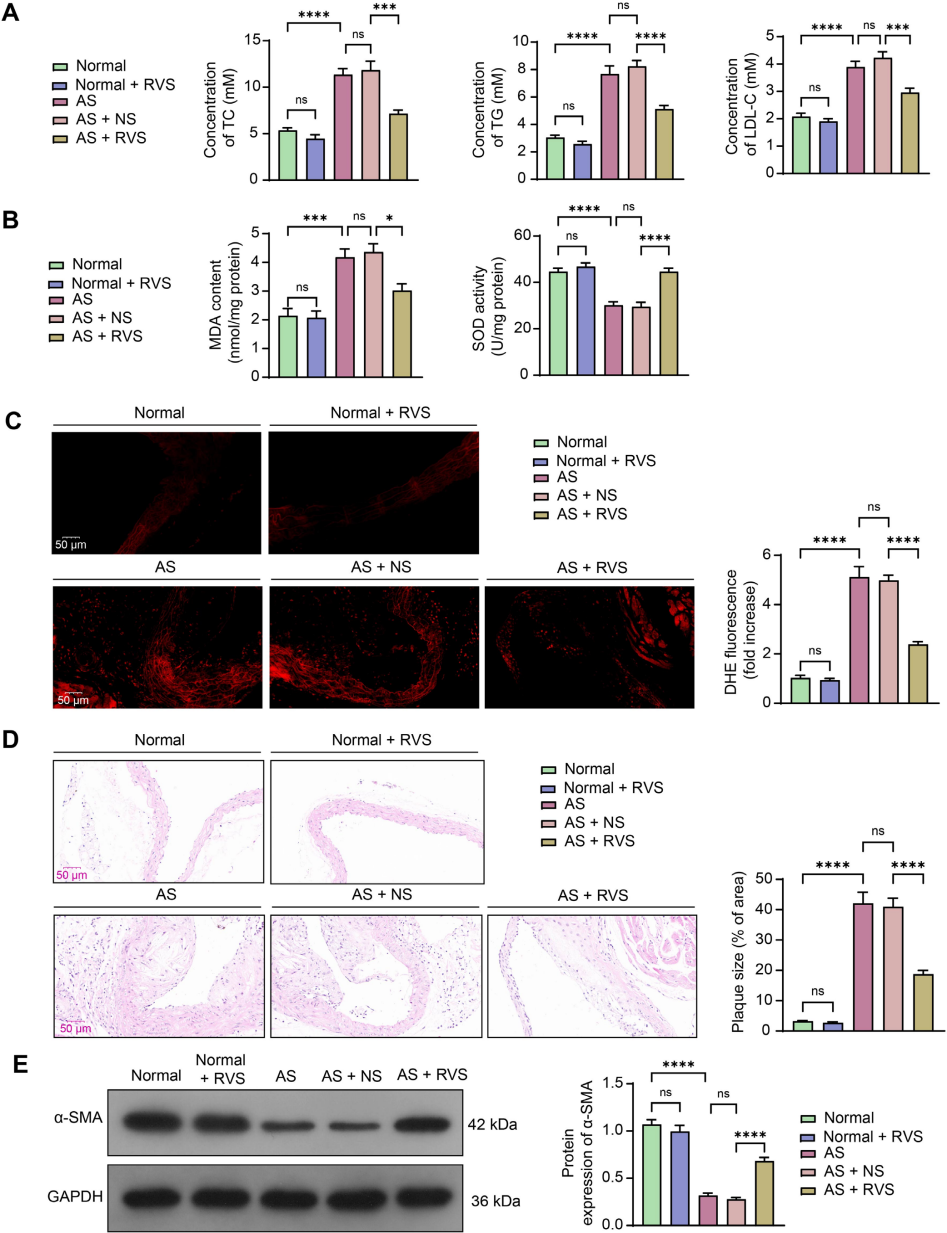
RVS treatment ameliorates AS‐associated symptoms in mice. ApoE^−/−^ mice were fed an HFD to generate a model of AS, and wild‐type mice fed a normal diet served as controls. (A) serum levels of TC, TG, and LDL‐C in mouse serum samples determined; (B) MDA content and SOD activity in the mouse aortic tissues determined using MDA and SOD assay kits, respectively; (C) ROS level in the mouse aortic tissues determined using DHE staining; (D) plaque formation in the tissue sections determined using HE staining; (E) protein expression of α‐SMA in the aortic tissue sections determined using western blot analysis. Each group contained 5 mice. Differences were compared by ANOVA. *p < 0.05, ***p < 0.001, ****p < 0.0001.

To verify the therapeutic effects of RVS on AS, a mouse model of AS was constructed by feeding ApoE^−/−^ mice an HFD. Compared to the Normal group, the use of RVS did not have a significant effect on serum lipid levels, which is consistent with a previous report [17]. Mice in the AS group showed increased serum levels of TC, TG, and LDL‐C, and combined RVS reversed this trend (Figure 1A). Using MDA and SOD assay kits, we found that MDA content was increased and SOD activity was decreased in the serum of modeled mice, and these trends were reversed by the RVS treatment as well (Figure 1B). DHE staining showed that the modeled mice exhibited higher levels of ROS in the aortic tissue sections, which were alleviated by the RVS treatment (Figure 1C). However, RVS did not have a significant effect on oxidative stress in mice in the Normal group (Figure 1B,C). HE staining showed that mice in the Normal group had normal aortic structures with no thickening of the intima, intact elastic fiber structure, and no AS plaques formed. However, mice in the AS group exhibited typical AS plaques in the vessels, with ruptured elastic fibers and widened subendothelial gaps (Figure 1D). Moreover, the RVS treatment was found to enhance the protein expression of α‐SMA, a marker of VSMCs, in the aortic tissues of mice, which was found to be reduced in modeled mice (Figure 1E). The above results confirmed that RVS has therapeutic effects on AS and excellent safety in normal mice.

### 3.2 RVS Alleviates Oxidative Stress and Proliferation of Ox‐LDL‐Induced HVSMCs by Upregulating RCOR1

**FIGURE 2:**
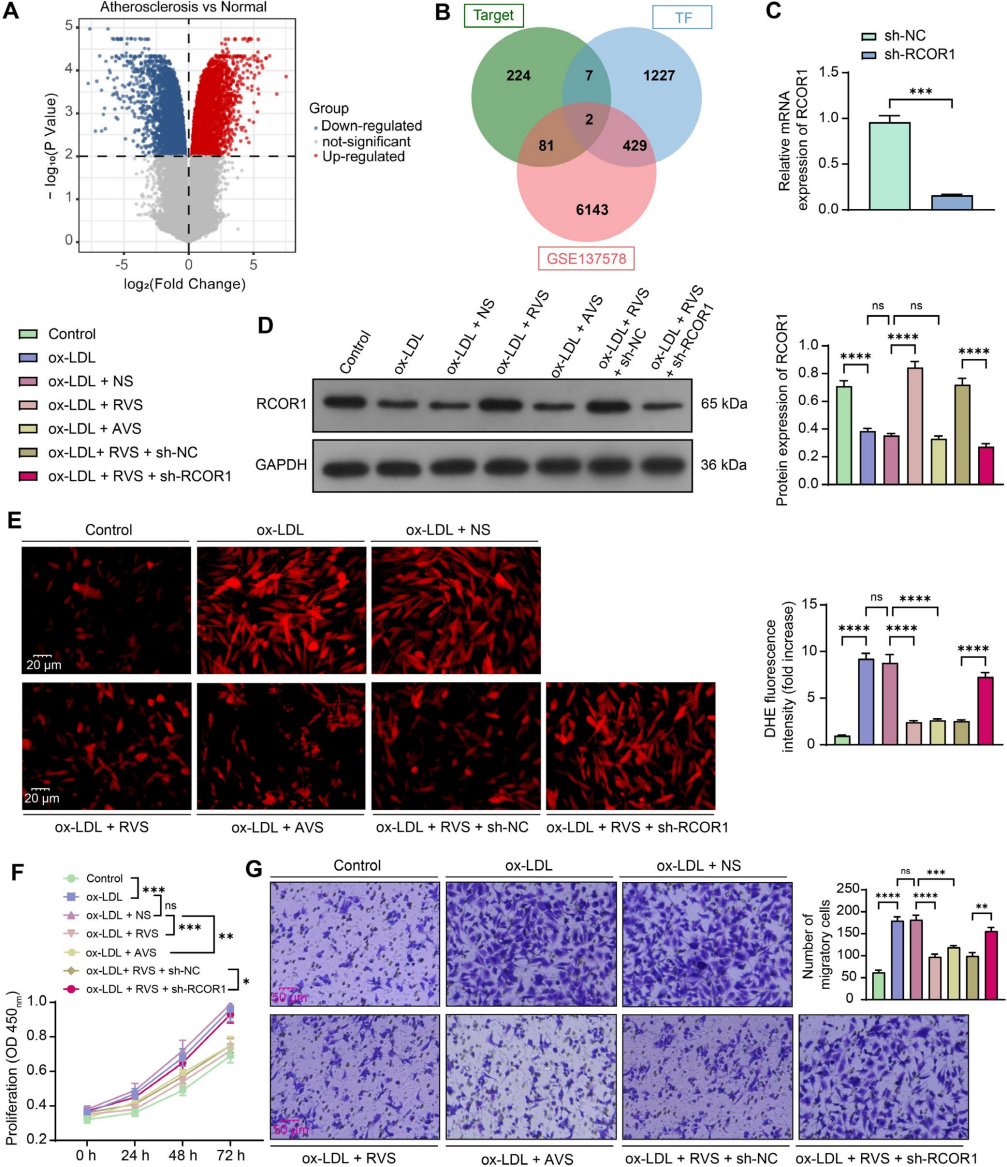
RVS alleviates oxidative stress and proliferation of ox‐LDL‐induced HVSMCs by upregulating RCOR1. (A) DEGs between AS and normal tissue samples analyzed using the GSE137578 dataset. (B) intersections between RVS targets predicted using the ChEMBL system, significant DEGs, and human transcription factors. (C) RCOR1 mRNA expression in HVSMCs following infection of sh‐RCOR1 determined using RT‐qPCR; HVSMCs, with or without transfection, were subjected to RVS incubation and the subsequent ox‐LDL treatment. (D) protein level of RCOR1 in cells determined using WB analysis. (E) ROS levels in cells determined using DHE staining. (F) proliferation of cells determined using CCK‐8 assays; (G) migration of cells determined using Transwell assays. Three independent experiments were performed. Differences were determined using ANOVA. *p < 0.05, **p < 0.01, ***p < 0.001, ****p < 0.0001.

To analyze the functional machinery of RVS, we predicted its molecular targets using the ChEMBL database (https://www.ebi.ac.uk/chembl/). Additionally, we analyzed differentially expressed genes (DEGs) between ox‐LDL‐treated and untreated cells using the GSE137578 dataset. Significant DEGs were identified under adj. *p*‐value < 0.01 (Figure 2A). Furthermore, we probed human transcription factors from the HumanTFDB system. An intersection analysis was performed to analyze intersections between predicted RVS targets, significant DEGs, and human transcription factors, leading to the identification of PPARG and RCOR1 (Figure 2B). PPARG has been demonstrated to be implicated in the ox‐LDL‐induced formation of foam cells in AS [18], while RCOR1's function in the progression of AS remains to be further elucidated. RCOR1 exhibits a low‐expression pattern in AS samples in the GSE137578 dataset (Log_2_ Foldchange = −0.405). Interestingly, RCOR1 has been demonstrated to exert anti‐oxidative properties in neurons [19]. Subsequently, we aimed to investigate the roles of RVS and RCOR1 in activity and oxidative stress in HVSMCs.

Stable HVSMCs with RCOR1 knockdown were screened using shRNA lentivirus, and RT‐qPCR validated the successful knockdown of RCOR1 expression in cells (Figure 2C). HVSMCs, with or without transfection, were subjected to RVS or positive control AVS incubation and the subsequent ox‐LDL treatment. Importantly, the RCOR1 protein levels were decreased by ox‐LDL treatment, increased in the presence of RVS pre‐treatment, and decreased upon sh‐RCOR1 transfection (Figure 2D). However, there was no significant change in RCOR1 expression in the ox‐LDL + AVS group (Figure 2D), indicating that RCOR1 is a specific target protein of RVS. The ROS levels in cells were upregulated by ox‐LDL stimulation, reduced by RVS or AVS treatments, and rescued upon RCOR1 silencing in the presence of RVS (Figure 2E). The changes in ROS levels paralleled the proliferation and migration activities of cells, which were promoted by ox‐LDL, blocked by RVS or AVS treatments, and rescued in the presence of RCOR1 silencing, according to the CCK‐8 (Figure 2F) and Transwell assays (Figure 2G).

### 3.3 Knockdown of RCOR1 Negates the Treatment Effect of RVS on Mice

**FIGURE 3:**
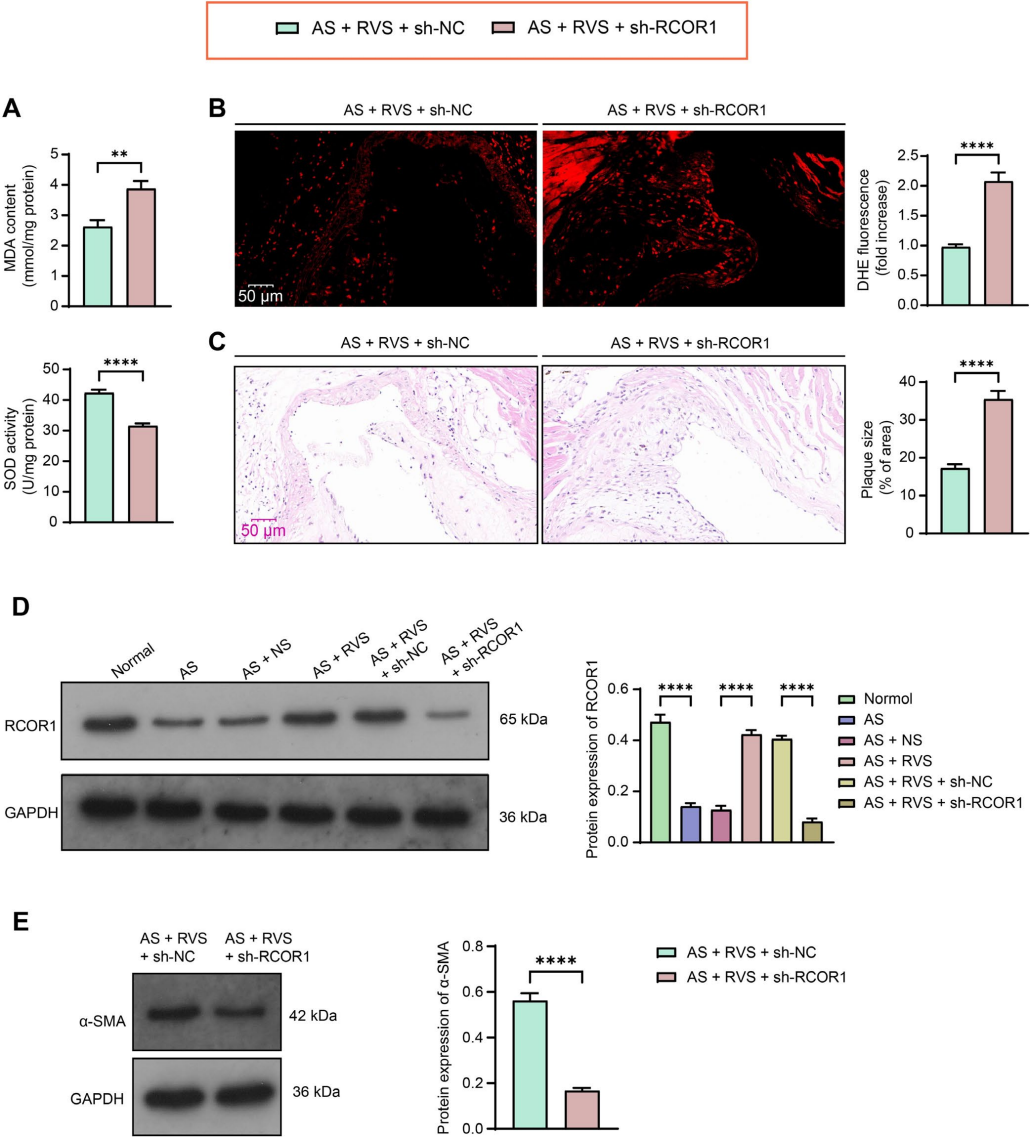
Knockdown of RCOR1 negates the treatment effect of RVS on mice. Modeled mice were administered adenovirus‐carried sh‐RCOR1, accompanied by RVS treatment. (A) MDA content and SOD activity in the mouse aortic tissues determined using MDA and SOD assay kits, respectively. (B) ROS level in the mouse aortic tissues determined using DHE staining. (C) plaque formation in the tissue sections determined using HE staining. (D) the protein expression of RCOR1 and α‐SMA in the aortic tissue sections determined using western blot analysis. Each group contained 5 mice. Differences were compared by the unpaired *t* test. **p < 0.01, ****p < 0.0001.

To verify the biological function of RCOR1 in the pathogenesis of AS, adenovirus‐carried sh‐RCOR1 was introduced into mice. The sh‐RCOR1 administration significantly increased MDA levels while reducing SOD activity in the mouse aorta (Figure 3A). The DHE staining revealed that the ROS levels in tissues were significantly elevated by RCOR1 knockdown as well (Figure 3B). The plaque formation in the mouse aortic tissue sections, initially improved by RVS treatment, was aggravated upon RCOR1 knockdown (Figure 3C). Additionally, the protein expression of RCOR1 and α‐SMA in the aortic tissue sections was weakened following sh‐RCOR1 administration (Figure 3D,E).

### 3.4 RCOR1 Loss Leads to Transcriptional Activation of C10ORF10

**FIGURE 4:**
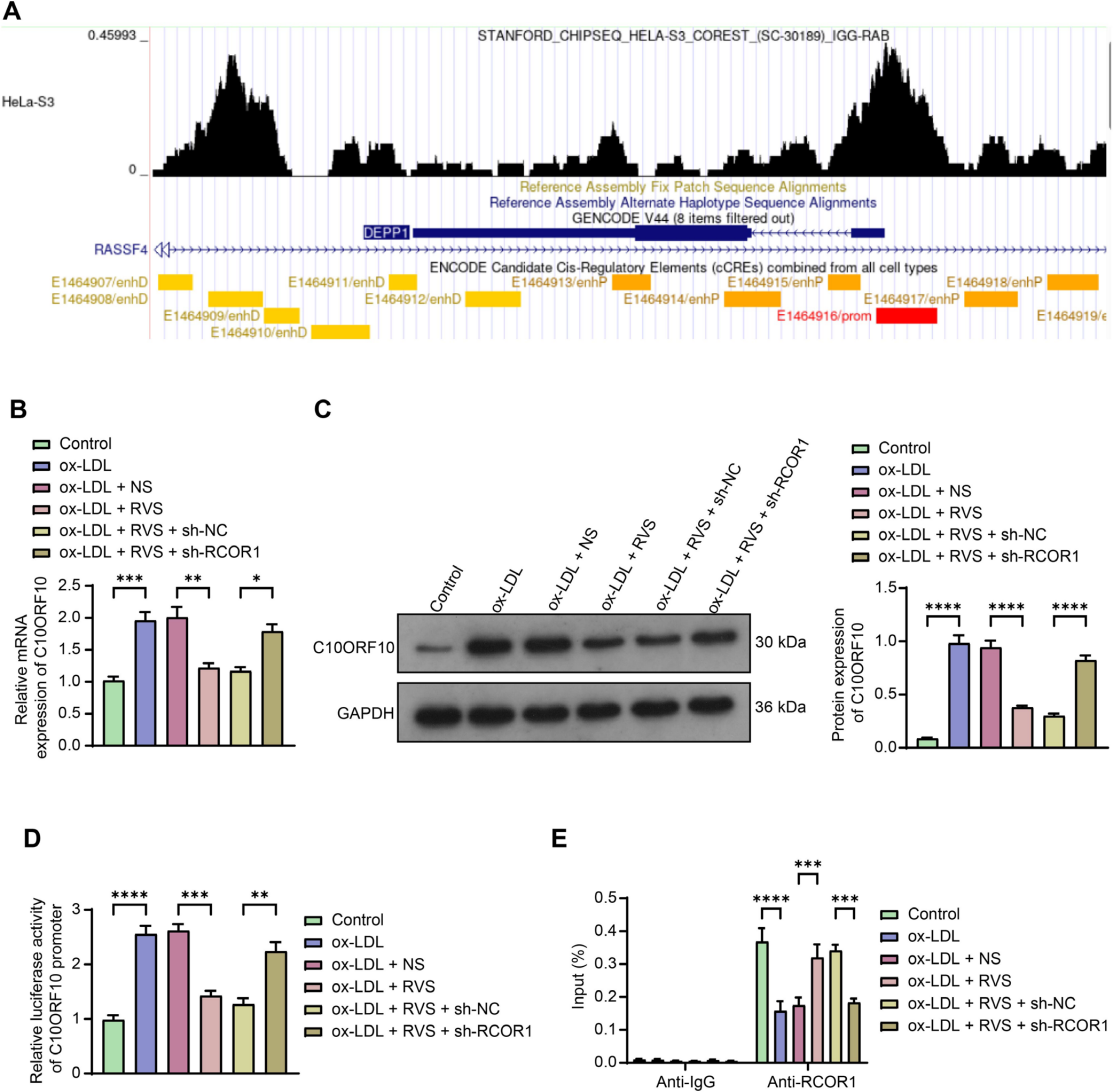
RCOR1 loss leads to transcriptional activation of C10ORF10. (A) binding peaks of RCOR1 near the C10ORF10 promoter region (chr10:44978762–44979110) predicted using the ChIP‐Seq data from the Cistrome database. (B, C) mRNA (B) and protein (C) levels of C10ORF10 in differentially treated HVMSCs determined using RT‐qPCR and WB analysis, respectively. (D) luciferase activity of the reporter vector containing the C10ORF10 promoter sequence in differentially treated HVMSCs determined using dual‐luciferase reporter gene assays. (E) binding between RCOR1 and the C10ORF10 promoter determined using ChIP‐qPCR assay. Three independent experiments were performed. Differences were determined using ANOVA (B–E). *p < 0.05, **p < 0.01, ***p < 0.001, ****p < 0.0001.

To investigate the downstream mechanism of RCOR1, DEGs obtained from GSE137578 were further screened. Given that RCOR1 is known as a potent transcription repressor [13] and the fact that it is poorly expressed in the context of AS, we focused on genes aberrantly highly expressed in AS (LogFC > 0). Notably, C10ORF10, the gene with the greatest degree of upregulation in AS in the GSE137578 dataset (Log2Fold change = 7.52), was predicted as a promising target of RCOR1. The ChIP‐Seq data from the Cistrome database (http://cistrome.org/db/#/) suggested significant binding peaks of RCOR1 near the C10ORF10 promoter region (chr10: 44978762–44979110) (Figure 4A). RT‐qPCR and WB analysis revealed that the mRNA and protein levels of C10ORF10 in HVSMCs were increased upon ox‐LDL treatment, decreased after RVS treatment, but restored following RCOR1 silencing (Figure 4B,C). Parallelly, the dual‐luciferase reporter assay demonstrated that the luciferase activity of the reporter vector containing the C10ORF10 promoter sequence was increased in cells after ox‐LDL treatment, reduced following RVS treatment, and increased after RCOR1 knockdown (Figure 4D). Furthermore, the ChIP assay revealed that the binding between RCOR1 and the C10ORF10 promoter in HVSMCs was reduced upon ox‐LDL treatment, increased following RVS treatment, and reduced again following RCOR1 silencing (Figure 4E).

### 3.5 Overexpression of C10ORF10 Counteracts the Effects of RVS On Ox‐LDL‐Treated HVSMCs

**FIGURE 5:**
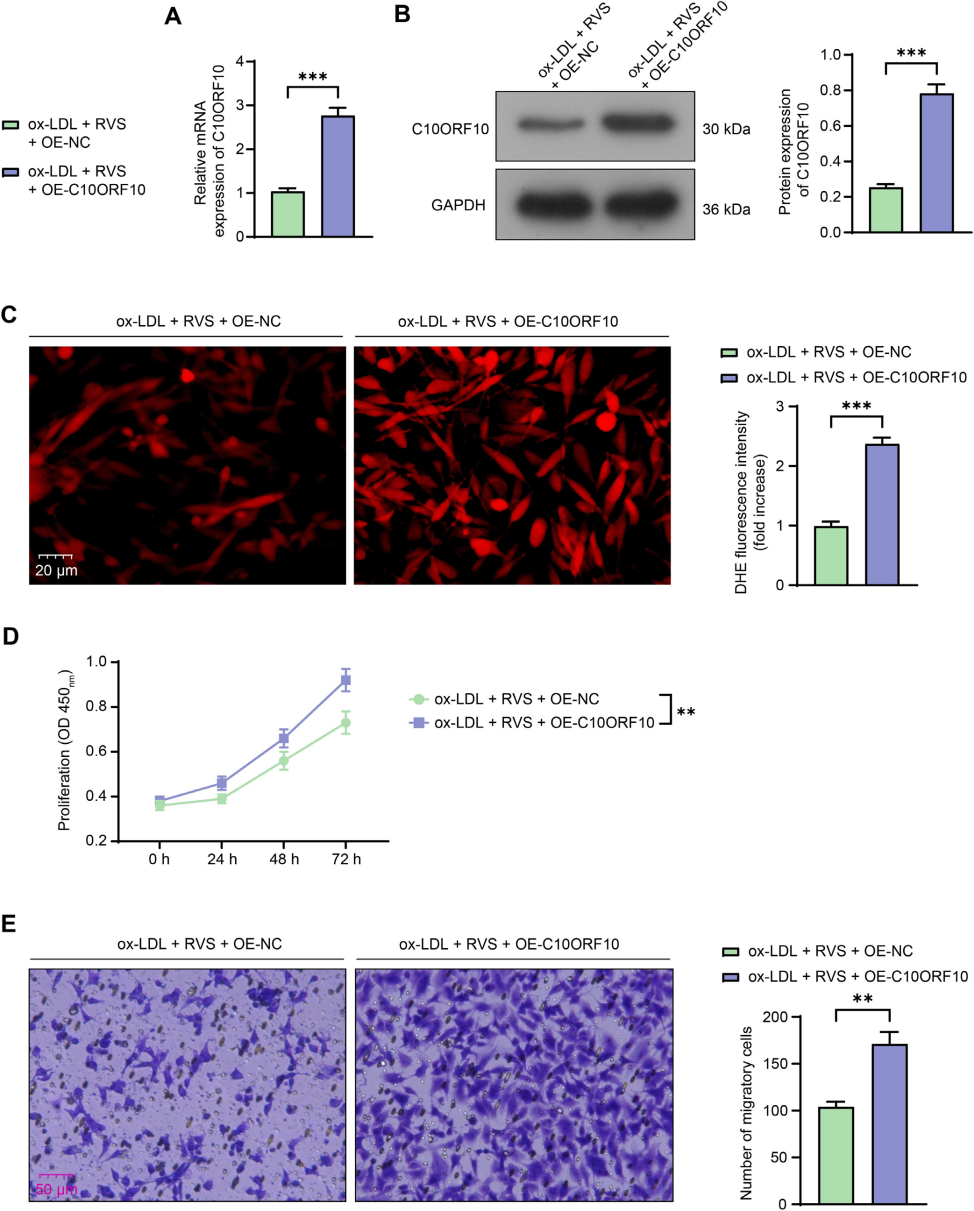
Overexpression of C10ORF10 counteracts the effects of RVS on ox‐LDL‐treated HVSMCs. HVSMCs were transfected with OE‐NC and OE‐C10ORF10. (A, B) mRNA (A) and protein (B) levels of C10ORF10 in HVSMCs were determined using RT‐qPCR and WB analysis; these cells were subjected to ox‐LDL and RVS treatments. (C) ROS levels in cells determined using DHE staining. (D) proliferation of cells determined using CCK‐8 assays. (E) migration of cells determined using Transwell assays. Three independent experiments were performed. Differences were determined using the unpaired *t* test (A, B, C, and E) or ANOVA (D). **p < 0.01, ***p < 0.001.

To examine whether C10ORF10 inhibition is implicated in the biological effects mediated by RVS, another two groups of HVSMCs were transfected with OE‐NC and OE‐C10ORF10, respectively. The successful upregulation of C10ORF10 in cells was determined by RT‐qPCR and WB analysis (Figure 5A,B). DHE staining revealed that the C10ORF10 overexpression significantly enhanced ROS levels in the HVSMCs in the presence of RVS treatment (Figure 5C). Additionally, the proliferation (Figure 5D) and migration (Figure 5E) of HVSMCs were restored following C10ORF10 upregulation.

### 3.6 Silencing of C10ORF10 Alleviates AS‐Like Symptoms in Mice Induced by RCOR1 Silencing

**FIGURE 6:**
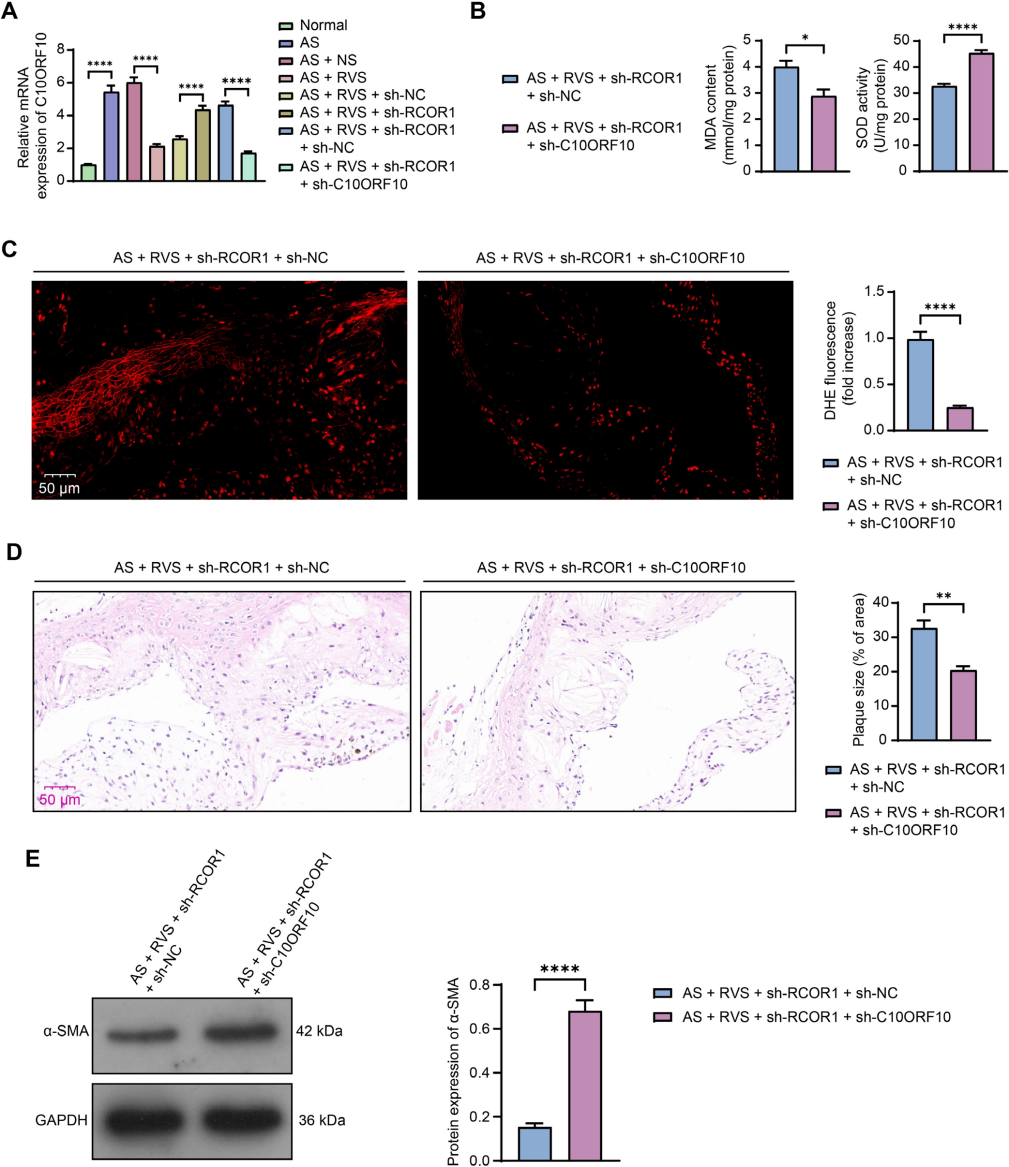
Silencing of C10ORF10 alleviates in mice induced by RCOR1 silencing. Modeled mice were administered adenovirus‐carried sh‐RCOR1 and the additional sh‐C10ORF10, accompanied by RVS treatment. (A) mRNA expression of C10ORF10 in mouse aortic tissues determined using RT‐qPCR. (B) MDA content and SOD activity in the mouse aortic tissues determined using MDA and SOD assay kits, respectively. (C) ROS level in the mouse aortic tissues determined using DHE staining. (D) plaque formation in the tissue sections determined using HE staining. (E) protein expression of α‐SMA in the aortic tissue sections determined using western blot analysis. Each group contained 5 mice. Differences were compared by the unpaired *t* test. *p < 0.05, **p < 0.01, ****p < 0.0001.

To validate the function of C10ORF10 in AS progression in vivo, mice treated with sh‐RCOR1 were additionally administered sh‐C10ORF10, accompanied by modeling and RVS treatments. RT‐qPCR results demonstrated that the mRNA expression of C10ORF10 was significantly increased in the aorta of AS mice, decreased following RVS administration, restored following RCOR1 silencing, and reduced by the additional administration of sh‐C10ORF10 (Figure 6A). The C10ORF10 knockdown significantly reduced the MDA content while increasing SOD activity in the mouse aortic tissues (Figure 6B), corresponding to a decrease in ROS levels (Figure 6C). Additionally, the plaque formation in the aortic tissue section aggravated upon RCOR1 silencing was ameliorated by the additional C10ORF10 knockdown (Figure 6D). Furthermore, the protein expression of α‐SMA in the aortic tissue sections was increased following C10ORF10 silencing (Figure 6E).

## 4 Discussion

Despite significant advancements in AS research, current treatments remain inadequate for effectively addressing the established disease [20]. Targeting the abnormal proliferation and migration of VSMCs has emerged as a promising strategy to curb the early stages of AS development [21]. Statins have been shown to possess pleiotropic effects that are not necessarily associated with their established lipid‐lowering properties [22]. In this study, we discovered a novel role of RVS in reducing oxidative stress and inhibiting the proliferation and migration of VSMCs by enhancing RCOR1‐mediated transcriptional repression of C10ORF10.

RVS is well‐known for its ability to inhibit HMG‐CoA reductase, the enzyme responsible for converting HMG‐CoA into mevalonate, a key precursor in cholesterol synthesis [10]. RVS has demonstrated the ability to reduce vascular morbidity and mortality, as well as to regress AS, either when used alone [23] or in combination with other regimens [11, 24]. On a molecular level, RVS has been shown to inhibit the expression of the transcription factor BTB and CNC homology 1, thereby protecting against AS [25]. As anticipated, we found that RVS treatment at 10 mg/kg/day significantly alleviated serum levels of TC, TG, and LDL‐C in ApoE^−/−^ mice fed an HFD, accompanied by a reduction in ROS levels and other oxidative stress markers in the aortic tissues and an amelioration in plaque formation. These findings partly coincide with the findings by Yu et al. [11]. Additionally, they found that RVS also suppressed oxidative stress in ox‐LDL‐stimulated human umbilical vein endothelial cells in vitro [11]. Here, we focused on the treatment of RVS on HVMSCs as they are the most abundant cells in vessels and major contributors to the early development of AS [21]. The expression of α‐SMA, a marker of contractile VSMCs [26], was decreased in the aorta of AS mice but restored following RVS treatment, indicating a suppressive role of RVS on the synthetic state switching of VSMCs. It has been posited that the process of cholesterol depletion, induced by the administration of statins, may play a pivotal role in the orchestration of VSMC migration [27]. Consistently, in vitro, the RVS treatment also significantly weakened ox‐LDL‐induced oxidative stress in HVSMCs, leading to a significant reduction in the proliferation and migration of these cells.

Here, we analyzed the downstream targets implicated in RVS's effects. RCOR1 was identified as a promising target for showing a reduction in atherosclerotic samples according to the GSE137578 datasets. While the role of RCOR1 in VSMC phenotype change or AS progression has not been investigated, previous evidence has highlighted its anti‐oxidative properties in neurons [19]. Here, we verified that the expression of RCOR1 was decreased in the aortic tissue of atherosclerotic mice and ox‐LDL‐induced HVMSCs, with RVS treatment improving its protein levels. Furthermore, silencing of RCOR1 negated the AS‐ameliorating effects of RVS in mice and HVSMCs by restoring oxidative stress levels. This indicates that restoring the RCOR1 protein level represents an important mechanism in RVS's effects on reducing oxidative stress and blocking the activity of HVMSCs.

Regarding the downstream targets mediated by RCOR1, we identified C10ORF10 as a promising target of it. It has been demonstrated to be dysregulated in mouse tissues in response to hypoxia and oxidative stress [28]. Its overexpression contributed to inducing ROS generation and oxidative damage [29]. Here, we observed that the C10ORF10 expression was increased in the aorta of AS mice, decreased following RVS treatment, but restored following RCOR1 knockdown. Its overexpression diminished the effects of RVS in vitro, while its knockdown alleviated AS induced by RCOR1 silencing. These effects indicate that the anti‐oxidative and AS‐ameliorating effects of RCOR1 were achieved, at least in part, through the suppression of C10ORF10.

Our study has some limitations. First, the dose for animal studies (10 mg/kg/day) is a supra‐therapeutic preclinical dose, and future work will titrate lower doses to better align with clinical regimens. In addition, we acknowledge that a single dose cannot define the minimal effective or maximal tolerated dose in vivo. We are planning to perform follow‐up experiments using at least three doses.

## 5 Conclusion

**FIGURE 7:**
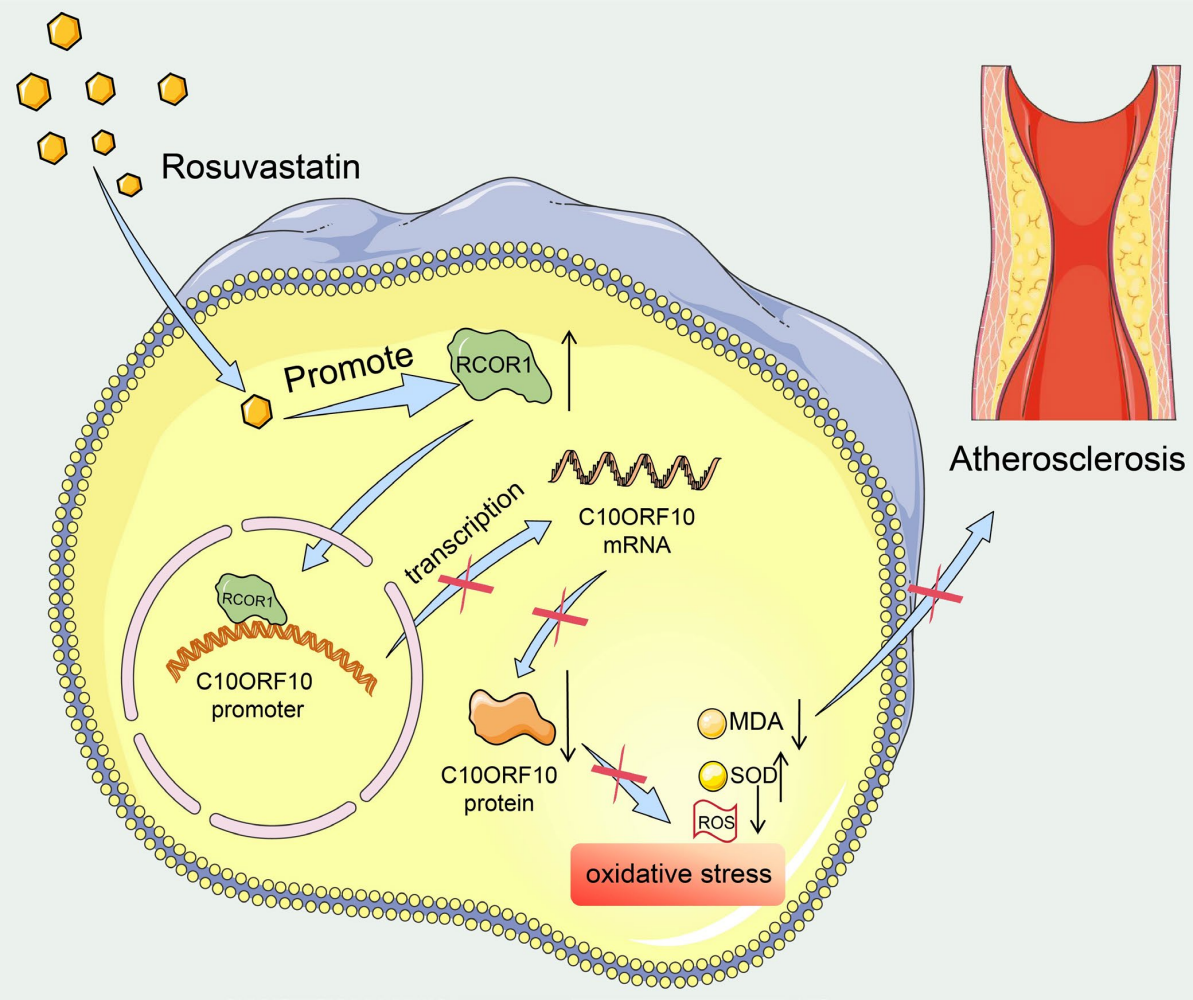
Schematic illustration. RVS inhibits oxidative stress and alleviates AS in mice by promoting the protein expression of RCOR1 and inhibiting the transcription of C10ORF10.

In summary, this study demonstrates that RVS alleviates oxidative stress and reduces atherosclerotic plaque formation by reducing the synthetic phenotype switching of VSMCs, which was achieved through RCOR1‐mediated transcriptional repression of C10OR10 (Figure 7).

## Conflicts of Interest

The authors declare no conflicts of interest.

## Data Availability

The data that support the findings of this study are available on request from the corresponding author. The data are not publicly available due to privacy or ethical restrictions.
